# Chrysanthemum *DgWRKY2* Gene Enhances Tolerance to Salt Stress in Transgenic Chrysanthemum

**DOI:** 10.3390/ijms19072062

**Published:** 2018-07-16

**Authors:** Ling He, Yin-Huan Wu, Qian Zhao, Bei Wang, Qing-Lin Liu, Lei Zhang

**Affiliations:** Department of Ornamental Horticulture, Sichuan Agricultural University, 211 Huimin Road, Wenjiang District, Chengdu 611130, Sichuan, China; heling@stu.sicau.edu.cn (L.H.); s20141825@sicau.edu.cn (Y.-H.W.); s20167109@stu.sicau.edu.cn (Q.Z.); s20167108@stu.sicau.edu.cn (B.W.); 14069@sicau.edu.cn (L.Z.)

**Keywords:** transgenic chrysanthemum, WRKY transcription factor, salt stress, gene expression, *DgWRKY2*

## Abstract

WRKY transcription factors (TFs) play a vital part in coping with different stresses. In this study, *DgWRKY2* was isolated from *Dendranthema grandiflorum*. The gene encodes a 325 amino acid protein, belonging to the group II WRKY family, and contains one typical WRKY domain (WRKYGQK) and a zinc finger motif (C-X4-5-C-X22-23-H-X1-H). Overexpression of *DgWRKY2* in chrysanthemum enhanced tolerance to high-salt stress compared to the wild type (WT). In addition, the activities of antioxidant enzymes (superoxide dismutase (SOD), peroxidase (POD), catalase (*CAT*)), proline content, soluble sugar content, soluble protein content, and chlorophyll content of transgenic chrysanthemum, as well as the survival rate of the transgenic lines, were on average higher than that of the WT. On the contrary, hydrogen peroxide (H_2_O_2_), superoxide anion (O_2_^−^), and malondialdehyde (MDA) accumulation decreased compared to WT. Expression of the stress-related genes *DgCAT*, *DgAPX*, *DgZnSOD*, *DgP5CS*, *DgDREB1A*, and *DgDREB2A* was increased in the *DgWRKY2* transgenic chrysanthemum compared with their expression in the WT. In conclusion, our results indicate that *DgWRKY2* confers salt tolerance to transgenic chrysanthemum by enhancing antioxidant and osmotic adjustment. Therefore, this study suggests that *DgWRKY2* could be used as a reserve gene for salt-tolerant plant breeding.

## 1. Introduction

High-salt stress is one of the most important factors that seriously affects and inhibits the growth and yield of plants [1]. Environmental stresses affect plant growth, causing plants to evolve mechanisms to face these challenges [2]. Under salt stress, transcription factors (TFs) can regulate the expression of multiple stress-related genes, which enhance tolerance to salt compared with the activity of a functional gene [3]. These genes are involved in the salt stress response in plants, forming a complex regulatory network [4]. Therefore, by using transcription factors, the plants’ resistance can be improved.

WRKYs are a massive TF family, dominating the genetic transcription of plants. WRKY was named after the highly conserved sequence motif WRKYGQK. The WRKY proteins are divided into 3 types: class I contains two conserved WRKY domains and a zinc finger structure C-X4-5-C-X22-23-H-X1-H; class II contains one conserved WRKY domain and the same zinc finger structure, and most of the WRKY proteins found to date are this type; there is one conserved domain in class III, the zinc finger structure C-X7-C-X22-23-H-X1-C [5]. Overexpression of genes is a commonly used method to study gene function. Many studies had shown that WRKY TFs played a vital role in the physiological processes of plants [6,7,8,9]. It has also been proved that overexpression of some WRKY genes successfully increase plant tolerance to abiotic stress. *TaWRKY93* may increase salinity tolerance by enhancing osmotic adjustment, maintaining membrane stability, and increasing transcription of stress-related genes [10]. During salt treatment, *NbWRKY79* enhanced the tolerance of the transgenic plants to oxidant stress. Therefore, it increased the salt tolerance of *Nicotiana benthamiana* [11]. *RtWRKY1* conferred tolerance to salt stress in transgenic *Arabidopsis* by regulating plant growth, osmotic balance, Na^+^/K^+^ homeostasis, and the antioxidant system [12]. *VvWRKY30* increased salt resistance by regulating reactive oxygen species (ROS)-scavenging activity and the accumulation of osmoticum [13].

Physiological traits are important indicative indexes of botanical abiotic resistance. Plants produce ROS in the body under environmental pressures, including accumulation of superoxide anions (O_2_^−^), hydroxyl ions (OH^−^), hydroxyl radicals (-OH), hydrogen peroxide (H_2_O_2_), and other types. These species not only lead to membrane lipid peroxidation of plant cells, affecting the redox state of the protein, but also cause oxidative damage to nucleic acids [14]. The plant antioxidant defense system consists of a variety of enzymes (superoxide dismutase (SOD), peroxidase (POD), catalase (*CAT*), ascorbate peroxidase (*APX*), etc.), which act as active oxygen scavengers in plants [15]. Much evidence has shown that the production and removal of ROS are closely related to the mechanism of salt tolerance [16,17]. As penetrating agents, soluble sugar, soluble protein, and proline maintain osmotic balance together. Under salt stress, transcription factors may participate in the regulation of the expression of many salt tolerance-related functional genes, so as to obtain stronger stress resistance than can be imparted by functional genes. The key genes encoding antioxidant enzymes (*Cu/ZnSOD*, *CAT*, *APX*, etc.) can increase the efficiency of ROS elimination in plant cells, so much so that the plant’s tolerance to abiotic stresses is improved [18,19,20]. The proline synthase gene (*P5CS*) can effectively increase the tolerance of transgenic plants to osmotic stress [21]. The DREB (dehydration-responsive element binding proteins) transcription factor can specifically bind to the DRE *cis*-acting element or the core sequence with the DRE element (CCGAC), regulate the expression of stress-related genes, and mediate the transmission of abiotic stress signals [22,23,24,25].

Chrysanthemums are cut flower with high economic benefits and appreciable value, but it is sensitive to salinity, which can cause slow growth, plant chlorosis, and even death [26]. In a previous study, we obtained a database of the chrysanthemum transcriptome in response to salinity conditions by using high-throughput sequencing [27]. A large number of salt-induced transcripts were found in the data, especially from the WRKY family. Previously, we identified four WRKY genes (*DgWRKY1*, *DgWRKY3*, *DgWRKY4*, and *DgWRKY5*) and demonstrated that they can increase the salt tolerance of tobacco or chrysanthemum [28,29,30,31]. In order to analyze the WRKY family in chrysanthemum from multiple angles to complement our information, the salt stress-related gene *DgWRKY2* was isolated from chrysanthemum. This study investigated the importance of *DgWRKY2* as a transcription regulator under salt stress.

## 2. Results

### 2.1. Isolation and Characterization of DgWRKY2

**Figure 1:**
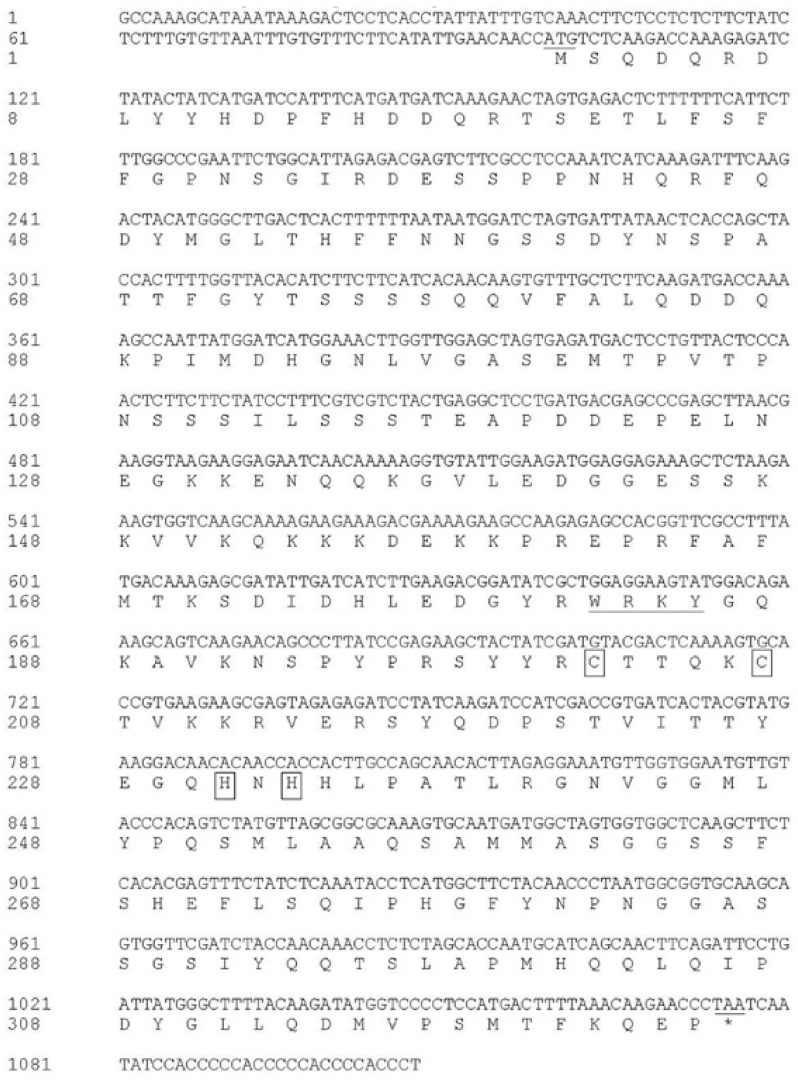
The nucleotide sequence and the deduced amino acid sequence of *DgWRKY2*. The WRKY domain is underlined. The cysteine and histidine in the zinc-finger motifs are boxed.

**Figure 2:**
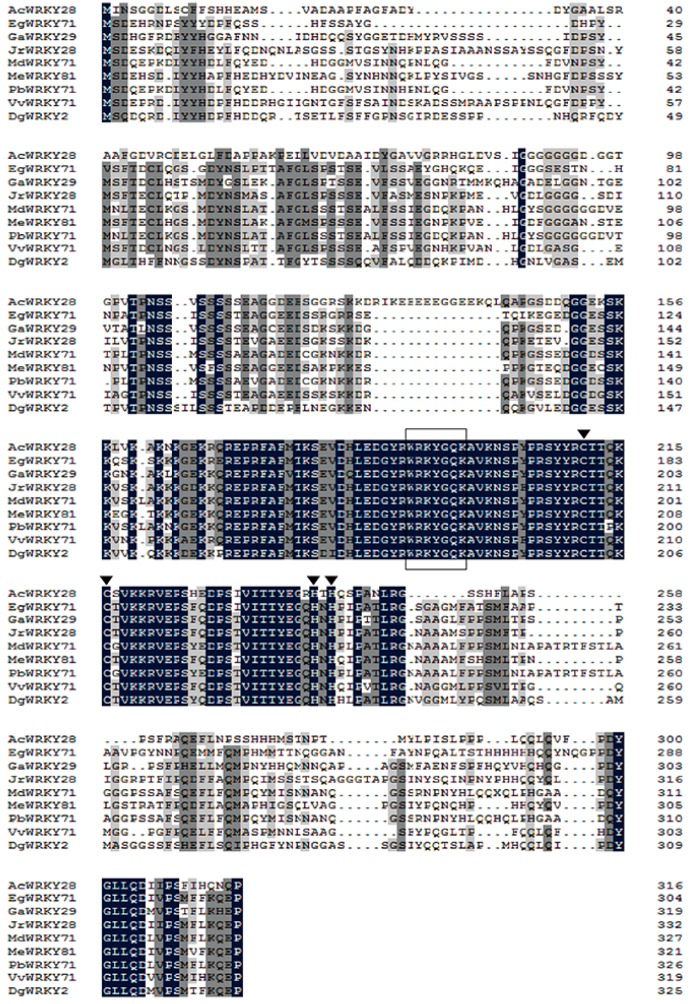
Comparison between the amino acid sequences deduced for the *DgWRKY2* gene. Amino acid residues conserved in all sequences are shaded in black, and those conserved in four sequences are shaded in light gray. The completely conserved WRKYGQK amino acids are boxed. The cysteine and histidine in zinc finger motifs are indicated by arrowheads (▼).

**Figure 3:**
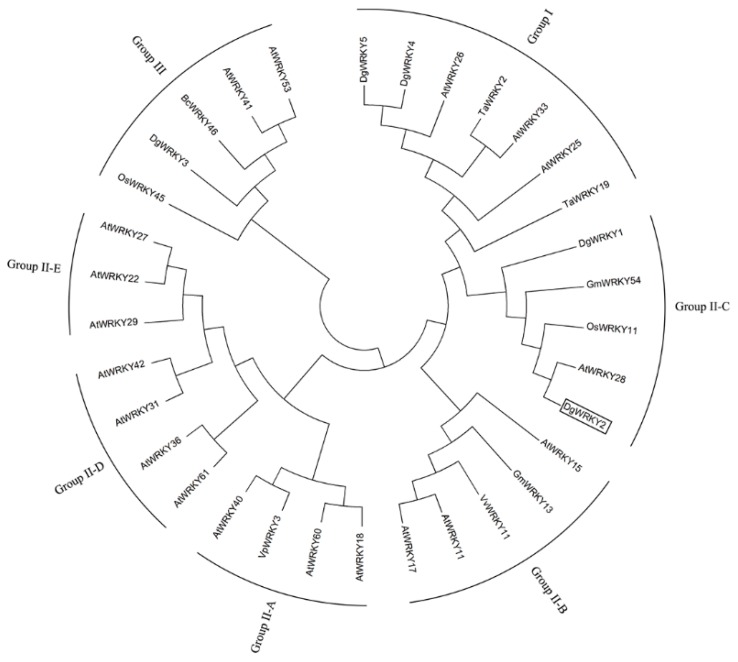
Phylogenetic tree analysis of the WRKY protein in different plants. The phylogenetic tree was drawn using the MEGA 5.0 program with the neighbor-joining method. *DgWRKY2* is boxed. The plant WRKY proteins used for the phylogenetic tree are as follows: *DgWRKY1* (KC153303), *DgWRKY3* (KC292215), *DgWRKY4*, *DgWRKY5* from *Dendranthema grandiflorum*; *AtWRKY11* (NP_849559), *AtWRKY15* (NP_179913.1), *AtWRKY17* (NP_565574.1), *AtWRKY18* (NP_567882), *AtWRKY22* (AEE81999), *AtWRKY25* (NP_180584), *AtWRKY26* (AAK28309), *AtWRKY27* (NP_568777), *AtWRKY28* (NP_193551), *AtWRKY29* (AEE84774), *AtWRKY31* (NP_567644), *AtWRKY33* (NP_181381), *AtWRKY36* (NP_564976), *AtWRKY40* (NP_178199), *AtWRKY41* (NP_192845), *AtWRKY42* (NP_192354), *AtWRKY53* (NP_194112), *AtWRKY60* (NP_180072), *AtWRKY61* (NP_173320) from *Arabidopsis thaliana*. *TaWRKY2* (EU665425), *TaWRKY19* (EU665430) from *Triticicum aestivum. GmWRKY13* (DQ322694), *GmWRKY54* (DQ322698) from *Glycine max. OsWRKY11* (AK108745), *OsWRKY45* (AY870611) from *Oryza sativa. VvWRKY11* (EC935078) from *Vitis vinifera. VpWRKY3* (JF500755) from *Vitis pseudoreticulata. BcWRKY46* (HM585284) from *Brassica campestris.*

*DgWRKY2* contained a complete open reading frame of 1107 bp, which encoded a protein of 325 amino acids with a calculated molecular mass of 36.55 kDa. The theoretical isoelectric point is PI = 6.66 (Figure 1). Multi-sequence alignment analysis of the amino acid sequences of *DgWRKY2* and eight other genes showed that *DgWRKY2* contains a WRKY domain and a zinc finger structure (C-X4-5-C-X22-23-H-X1-H). It was further confirmed that the cloned cDNA sequence was a WRKY transcription factor II family member (Figure 2). Phylogenetic analysis showed that *DgWRKY2* is most closely related to *AtWRKY28* from *Arabidopsis thaliana* (Figure 3).

### 2.2. Salt-Tolerance Analysis of DgWRKY2 Transgenic Chrysanthemum

**Figure 4:**
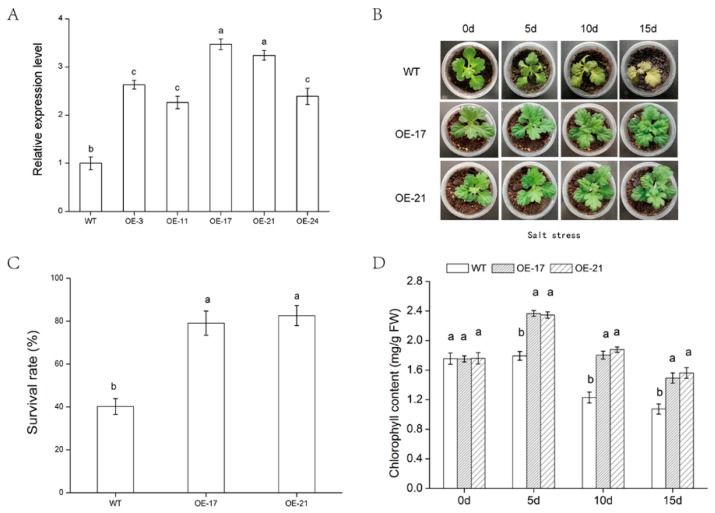
Expression of chrysanthemum in different strains under salt stress. (**A**) Relative expression level of *DgWRKY2* in transgenic chrysanthemums. The different normal letters indicate a significant difference at the 0.05 level among different strain lines, the same below; (**B**) comparison of transgenic plants and wild type plants after different periods under salt stress; (**C**) chrysanthemum survival statistics after recovery; (**D**) chlorophyll contents of chrysanthemum leave under salt stress.

To determine whether *DgWRKY2* overexpression enhanced salt tolerance, chrysanthemum transgenic lines with overexpressed *DgWRKY2* were produced by *Agrobacterium*-mediated transformation. *DgWRKY2* transcription levels in up in five transgenic lines (OE-3, OE-11, OE-17, OE-21 and OE-24) were detected by qRT-PCR (Figure 4A). We compared the salt stress tolerance between OE-17 and OE-21 transgenic chrysanthemum and the WT. Under normal growth conditions, the phenotypic differences were not significant. The growth rate was consistent. By contrast, under salt stress, wilting and yellowing of leaves of the WT plants were evident (Figure 4B). After the recovery period (2 weeks), the survival rate in the WT was 40.23%, while the survival rates in transgenic lines OE-17 and OE-21 were 79.07% and 82.60%, respectively (Figure 4C). The survival rate of transgenic chrysanthemums was significantly higher than that of the WT.

### 2.3. Analysis of Chlorophyll Content and under Salt Stress

Salt stress significantly inhibited plant photosynthesis [32]. The content of chlorophyll in the leaves of the WT decreased obviously at the 10th day, while reaching the minimum value at the 15th day. However, the chlorophyll content from the transgenic chrysanthemum lines OE-17 and OE-21 increased significantly, by 35% and 33% at the 5th day, and decreased gradually later on. In general, the decrease of chlorophyll content in transgenic chrysanthemum is lower than that of the WT (Figure 4D).

### 2.4. Accumulation of H_2_O_2_, O_2_^−^, and MDA in DgWRKY2 Transgenic Chrysanthemum under Salt Stress

**Figure 5:**
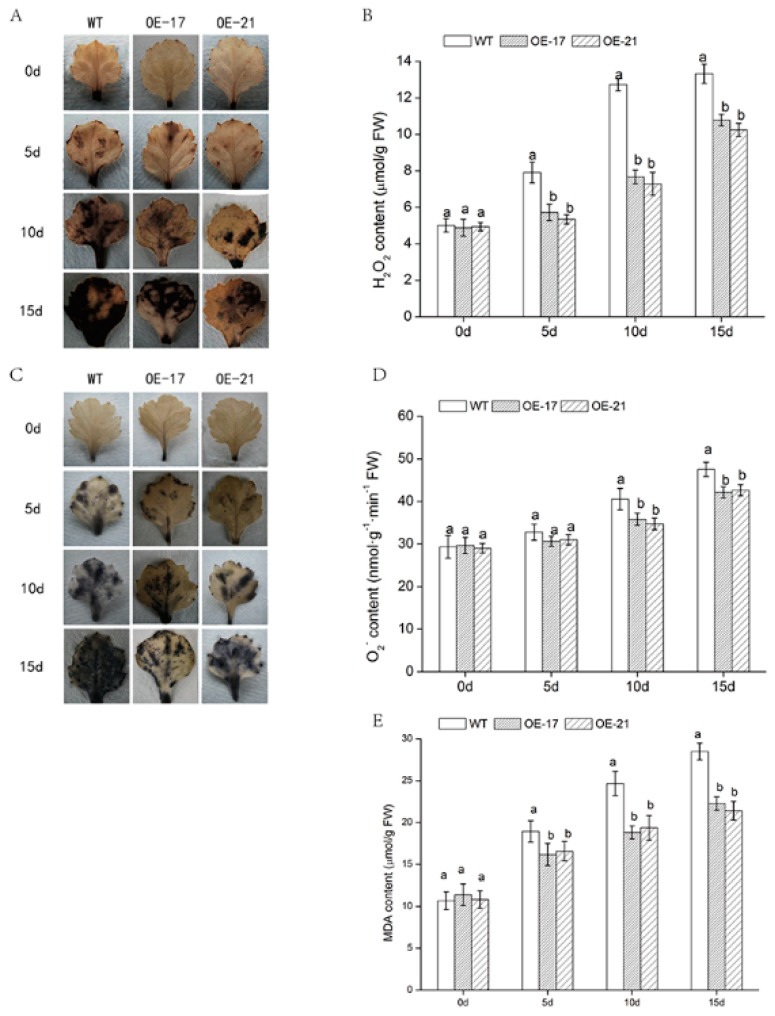
The levels of oxidative damage in WT and *DgWRKY2* overexpression lines of chrysanthemum were analyzed. (**A**) Diaminobenzidine (DAB) staining of chrysanthemum leaves during salt stress treatment; (**B**) changes in H_2_O_2_ content under salt stress; (**C**) nitroblue tetrazolium (NBT) staining of chrysanthemum leaves during salt stress treatment; (**D**) changes in O_2_^−^ content under salt stress; (**E**) changes in malondialdehyde (MDA) content of chrysanthemum leaves under salt stress. Data represent means and standard errors of three replicates. The different letters above the columns indicate significant differences (*p* < 0.05) according to Duncan’s multiple range test.

Reactive oxygen species in plant cells have a strong toxic effect. In order to study the effect of transgenic lines on the scavenging of reactive oxygen species, H_2_O_2_ and O_2_^−^ in different lines were investigated with DAB and NBT staining. Under normal circumstances, there was no significant difference in H_2_O_2_ and O_2_^−^ between the WT and two transgenic lines. After treatment with salt stress, the H_2_O_2_ content in each line increased significantly (Figure 5A,B). The contents of O_2_^−^ showed an upward trend with the increase of stress time (Figure 5C,D), but it was not as obvious as that of H_2_O_2_. Under salt stress, despite the rising trend, the accumulation of H_2_O_2_ and O_2_^−^ in the transgenic lines was much lower than that of the WT. These results indicate that the overexpression of *DgWRKY2* might regulate the activity of antioxidant protective enzymes, conferring greater tolerance to salt stress in transgenic plants. Similarly, under salt stress, the MDA accumulation level of overexpressed lines was apparently lower than that of the WT (Figure 5E). In all, these results provided strong evidence that the accumulation of ROS in *DgWRKY2* overexpression chrysanthemum was lower than that of WT under salt stress. Thus, *DgWRKY2* overexpression reduced the ROS level and alleviated the oxidant damage under salt stress.

### 2.5. Physiological Changes in DgWRKY2 Transgenic Chrysanthemum

**Figure 6:**
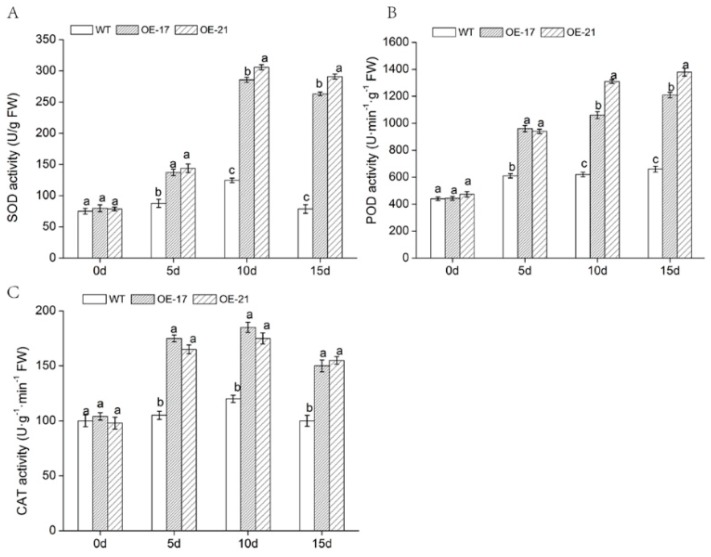
Changes in antioxidant enzyme activities of chrysanthemum leaves under salt stress. (**A**) Superoxide dismutase (SOD) activity under salt stress; (**B**) peroxidase (POD) activity under salt stress; (**C**) catalase (*CAT*) activity under salt stress. Data represent means and standard errors of three replicates. The different letters above the columns indicate significant differences (*p* < 0.05) according to Duncan’s multiple range test.

Antioxidant enzymes play an important part in botanical stress tolerance. We observed activities of SOD, POD, and *CAT* in the leaves of *DgWRKY2* lines and WT plants at different stages of treatment. Under normal growth conditions, the activities of these three enzymes had no obvious differences in any of the lines. Under salt treatment conditions, there was an increase in the WT and overexpressed lines. Moreover, compared with WT, these increases were extraordinarily greater in the overexpressed lines (Figure 6A–C). As a result, overexpression of *DgWRKY2* increases the antioxidant enzyme activity of transgenic chrysanthemum to counteract injury from ROS. Thus, this reduced oxidative damage.

**Figure 7:**
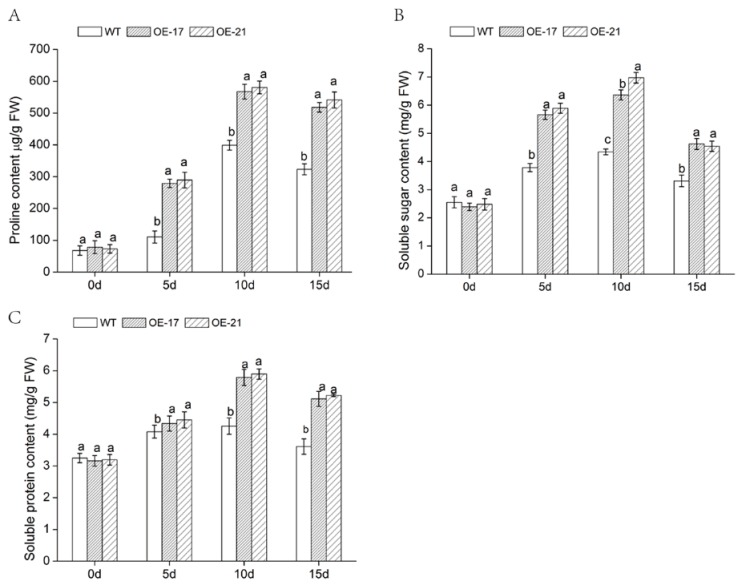
Changes in contents of osmotic adjustment substances of chrysanthemum leaves under salt stress. (**A**) Proline content under salt stress; (**B**) soluble sugar content under salt stress; (**C**) soluble protein content under salt stress. The different letters above the columns indicate significant differences (*p* < 0.05) according to Duncan’s multiple range test.

Osmotic adjustment is one of the most basic characteristics of plant salt tolerance, while proline is the most widely distributed compatible penetrant [33,34]. Under salt stress, we measured the proline content of transgenic lines and the WT in order to understand the osmoregulation ability of transgenic plants (Figure 7A). There was little difference in proline content between transgenic lines and WT under normal circumstances. By contrast, under salt stress, there was a remarkable increase in proline content for both. Nevertheless, the accumulation of proline in the transgenic lines was significantly higher than that of the WT under salt stress. These results indicate that *DgWRKY2* upregulated the accumulation of proline in the transgenic lines under salt stress.

Soluble proteins keep cells appropriately permeable and protect cells from dehydration, while stabilizing and protecting the structure and function of biological macromolecules [35]. We observed the content of soluble protein and of soluble sugar of these three lines under salt stress. In this environment, soluble protein and soluble sugar content of overexpressed lines increased significantly compared with the WT. (Figure 7B,C). The above data suggest that overexpression of *DgWRKY2* enhanced the osmoregulation ability of transgenic chrysanthemum while it increased its salt tolerance.

### 2.6. Expression of Abiotic Stress-Related Genes in DgWRKY2 Transformed Chrysanthemum

**Figure 8:**
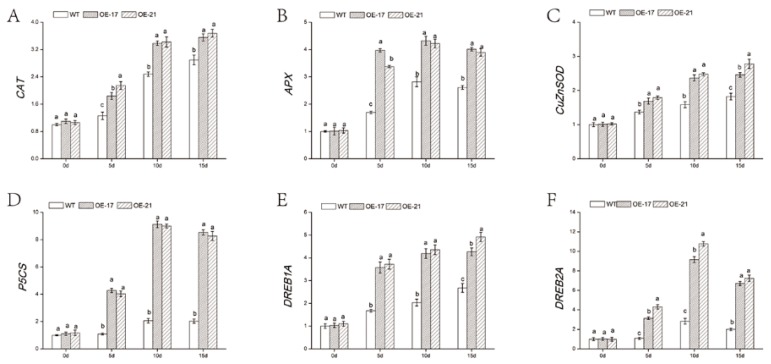
Expression of stress-related genes in wild type (WT) and overexpressed lines. (**A**) Expression analysis of *Cu/ZnSOD* under salt stress; (**B**) expression analysis of *CAT* under salt stress; (**C**) expression analysis of ascorbate peroxidase (*APX*) under salt stress; (**D**) expression analysis of *P5CS* in chrysanthemum under salt stress; (**E**) expression analysis of *DREB1A* under salt stress; (**F**) expression analysis of *DREB2A* under salt stress. Data represent means and standard errors of three replicates. The different letters above the columns indicate significant differences (*p* < 0.05) according to Duncan’s multiple range test.

In order to reveal the signal regulatory network of transgenic lines in the stress resistance process, we measured the expression of several functional genes involved in signal transduction pathways by qRT-PCR. Under standard circumstances, there was little difference in the expression of abiotic stress-response genes. When exposed to salt stress, the expression level of the gene encoding ROS-scavenging enzymes (*CAT*, *APX*, and *Cu/ZnSOD*) in the transgenic lines was much higher than in the WT (Figure 8A–C). Additionally, *P5CS*, a gene related to proline synthase, showed an expression level with a similar trend (Figure 8D). Furthermore, other genes, such as *DREB1A* and *DREB2A*, that are closely related to plant responses to environmental stresses, were all significantly upregulated in the overexpressed lines compared to the WT under salinity conditions (Figure 8E,F). Our data suggest that *DgWRKY2* overexpression could reduce osmotic pressure by clearing excess ROS and accumulating proline, thereby promoting salt tolerance.

## 3. Discussion

To date, the WRKY gene has been cloned from *Arabidopsis thaliana* [36], wheat [37,38], rice [39], soybean [40], chrysanthemum [28,29], birch [41], and other plants. It was confirmed that the WRKY gene is related to plant stress resistance. We isolated a new WRKY transcription factor—*DgWRKY2—*from chrysanthemum, and found it to be induced by salt stress. The deduced amino acid sequence of the *DgWRKY2* gene from this study contains one WRKY domain (WRKYGQK) and a zinc finger structure (C-X4-5-C-X22-23-H-X1-H), which could be considered part of the group II WRKY family.

The same group of WRKY proteins might have similar capabilities. Previous studies have shown that *GmWRKY54* might improve the salt and cold tolerance of plants through the regulation of *DREB2A* and STZ/Zat10 [40]. *OsWRKY11* overexpression increased rice drought tolerance [42]. The expression of *AtWRKY28* changed significantly under NaCl stress, indicating that *AtWRKY28* had much to do with adaptation to environmental stress [43]. In our previous study, an overexpression *DgWRKY1* tobacco line was more tolerant to salt stress than the WT [28]. *DgWRKY2* belongs to group II with *GmWRKY54*, *OsWRKY11*, *AtWRKY28*, and *DgWRKY1*, thus, we hypothesized that *DgWRKY2* has a positive effect on salt tolerance. In addition, the previous studies demonstrated that *DgWRKY3*, *DgWRKY4*, and *DgWRKY5* also played a positive regulatory role on salt stress [29,30,31]. *DgWRKY1* and *DgWRKY3* were only studied for their role in salt tolerance in tobacco, and the salt tolerance in chrysanthemum has yet to be studied. Previous studies have confirmed that *DgWRKY4* and *DgWRKY5* belong to the group III, and *DgWRKY2* in this study belongs to group II. The results of these studies showed that *DgWRKY4* and *DgWRKY5* imparted stronger salt tolerance than *DgWRKY2*. This is partly due to different groups playing different roles in the stress regulatory network. Additional work is needed to understand the mechanisms.

In this study, the *DgWRKY2* overexpression transgenic chrysanthemum was compared with the WT from physiological and biochemical aspects, and the function of *DgWRKY2* overexpression was verified. Chlorophyll content in chrysanthemum leaves continued to decrease in the late stage of salt stress. We speculated that ROS inhibited the photosynthesis of chrysanthemum [44]. However, chlorophyll content in the overexpressed lines was higher than that of the WT at respectively different salt stress stages. Increased ROS activity causes a great deal of physiological and metabolic changes in plants, enabling them to cope with environmental stress. In *CmWRKY17*-overexpressing plants, *CmWRKY17* altered the salinity sensitivity via regulation of ROS levels [45]. *NbWRKY79* was involved with the regulation of SOD, POD, *CAT*, and *APX* activities, which resulted in the suppression of ROS accumulation so that the plant could endure less oxidative damage under salt conditions [11]. *MsWRKY11* might reduce ROS levels and thus increase salt tolerance in soybean [46]. The activity of antioxidant enzymes SOD, POD, and *CAT* in *DgWRKY2* overexpression lines increased, and the activity of the enzymes was significantly higher than that of the WT at each stage of salt treatment. Moreover, the content of H_2_O_2_ and O_2_^−^ in transgenic chrysanthemum leaves was also lower than that of WT. The above results indicate that *DgWRKY2* overexpression could enhance plant antioxidant capacity by increasing the activities of SOD, POD, and *CAT*, thereby enhancing the salt tolerance of transgenic chrysanthemum.

Accumulation of MDA content can lead to membrane lipid peroxidation of plant cells, causing changes in the cell membrane structure and permeability, reducing cell function [47]. In contrast, proline prevents membrane lipid peroxidation, maintains normal cellular structure, and maintains a stable cell osmotic pressure [48]. Under salt treatment, compared with the WT, MDA content of the overexpressed lines was lower, but proline content was higher. The contents of soluble sugar and soluble protein in *DgWRKY2* overexpression lines were higher than those of WT. The results suggest that *DgWRKY2* might increase its salt tolerance by regulating the osmotic pressure of plant cells.

The expression of antioxidant genes (*Cu/ZnSOD*, *CAT*, and *APX*) was upregulated under salinity, which is consistent with physiological results. Under salt stress, the expression of antioxidant enzyme genes was significantly higher in *RtWRKY1*-overexpressed *Arabidopsis* than in the wild type [12]. The *P5CS* gene is associated with a proline-synthesizing enzyme in plants. When the expression of the *P5CS* gene was induced by environmental stress, the proline content in plants increased. Under salt stress, the expression of genes related to proline biosynthesis was upregulated in *VvWRKY30* transgenic lines compared with their expression in the WT [13]. These results show that transgenic plants exhibited increased expression levels of *P5CS* under stress conditions. The DREB gene belongs to the *AP2/EREBP* transcription factor family. These TFs are closely related to the response of plants to the environment [49,50]. In this study, *DREB1A* was upregulated to a greater extent in overexpressed lines than in WT, and *DREB2A* first increased and later decreased. Previous studies indicated that *OsDREB2A* might participate in abiotic stress by directly binding the DREB element to regulate the expression of downstream genes. Overexpression of *OsDREB2A* in soybean might be used to improve its tolerance to salt stress [51]. Cong found that overexpression of the *OjDREB* gene improved salt tolerance in tobacco plant [52]. These results suggest that enhanced salt tolerance was associated with the induction of downstream stress-related gene expression in *DgWRKY2* transgenic plants.

## 4. Materials and Methods

### 4.1. Plant Materials

The experimental material used for treatment is a wild-type chrysanthemum: *Dendranthema grandiforum*—‘Jinba’. All plant materials were provided by Sichuan Agricultural University, Chengdu, China. Chrysanthemum seedlings grew on MS culture medium (200 μL m^−2^ s^−1^, 16 h photoperiod, 25 °C/22 °C day/night temperature, and 70% relative humidity) for 20 days. Then, 20-day-old seedlings were planted in basins filled with a 1:1 mixture of peat and perlite, incubated for 3 days, and watered once daily (70% of field capacity). Seedlings at the six-leaf stage were harvested, frozen in liquid nitrogen immediately, and stored at −80 °C for RNA extraction.

### 4.2. Cloning of DgWRKY2 and Sequence Analysis

The RNA extraction of chrysanthemum leaves was performed by TRNzol reagent (Mylab, Beijing, China). The full-length cDNA of the *DgWRKY2* sequence was obtained by PCR (polymerase chain reaction) utilizing gene-specific primers (Table 1). The RACE reactions were carried out according to the manufacturer’s protocol (Invitrogen RACE cDNA amplification kit, Clontech, Mountain View, CA, USA). The fragment generated was cloned into pEASY-T1 Cloning Kit (Transgene Biotech, Beijing, China) and sequenced.

The sequence of *DgWRKY2* was analyzed by the National Center for Biotechnology Information (NCBI, http://www.ncbi.nlm.nih.gov/gorf/gorf.html) to obtain its open reading frame (ORF). Identification of protein domains and significant sites was performed with Motifscan (http://myhits.isb-sib.ch/cgi-bin/motif_scan). The phylogenetic tree was drawn with the MEGA 5.0 program (Sudhir Kumar, Arizona State University, Tempe, AZ, USA) using the neighbor-joining method.

### 4.3. Generation of Transgenic Chrysanthemum

The pEASY-WRKY2 cloning vector was constructed by TA cloning technology (The complementarity between the vector 3′-T overhangs and PCR product 3′-A overhangs allows direct ligation of Taq-amplified PCR products into the T-vector). The plasmid containing the pEASY-WRKY2 and pBI121 expression vectors were double digested with *Sac*I and *Xba*I to construct the *pBI121-DgWRKY2* expression vector. The fused construction of *pBI121-DgWRKY2* was transformed into the leaf disk of chrysanthemum by *Agrobacterium tumefaciens* (strain LBA4404) [53]. Callus induction from chrysanthemum was used to form seedlings [54]. The obtained *DgWRKY2* transgenic chrysanthemum lines (OE-17 and OE-21) were employed in subsequent experiments. The transgenic lines OE-17 and OE-21 were expanded for subsequent replication experiments.

### 4.4. Expression of DgWRKY2 under Salt Treatment

The method of RNA extraction is the same as above. Then RNA was used for first-strand cDNA synthesis with reverse transcriptase (TransScript II All-in-one First-Strand cDNA Synthesis SuperMix for PCR, Transgene, Beijing, China) according to the manufacturer’s protocol. Quantitative real-time PCR (qRT-PCR) was performed by SsoFast EvaGreen supermix (Bio-Rad, Hercules, CA, USA) and Bio-Rad CFX96TM detection system. The gene elongation factor 1α (*EF1α*) was used as a reference for quantitative expression analysis. A final 20 μL qPCR reaction mixture contained: 10 μL SsoFast EvaGreen supermix, 2 μL diluted cDNA sample, and 300 nM primers. Then, the reactions were incubated following the standard process: 1 cycle of 95 °C for 30 s, 40 cycles of 15 s at 95 °C and 30 s at 60 °C, and a single melting cycle from 65 to 95 °C. To avoid experimental errors, each reaction was repeated at least three times. To avoid variables and statistic error, a negative control group was set up, in which water supplanted the above solution. Relative expression levels were calculated by the 2^−ΔΔ*C*t^ method [55].

### 4.5. Salt Treatment of Transgenic Chrysanthemum and Stress Tolerance Assays

Two overexpressed lines (OE-17 and OE-21) and the WT of chrysanthemum, all 20 days old, were sown into a 1:1 mixture of peat and perlite, then cultured in a light incubator (200 μL m^−2^ s^−1^, 16 h photoperiod, 25 °C/22 °C day/night temperature, and 70% relative humidity). Soil-grown chrysanthemum seedlings at the six-leaf stage were irrigated with an increasing concentration of NaCl solution: 100 mm for 1–5 days (d), 200 mm for 6–10 days, and 400 mm for 11–15 days, using Chen as a reference [56]. Under salt stress, leaves 4–5 were harvested at 0, 5, 10, and 15 days for both physiological and molecular experiments. After a 2-week recovery, the surviving plants were collected to calculate the survival rate.

### 4.6. Determination of Physiological Indexes of Transgenic Chrysanthemum under Salt Stress

Activities of superoxide dismutase (SOD), peroxidase (POD), and catalase (*CAT*) were measured according to Li [57]. Malondialdehyde (MDA) content in chrysanthemum was measured according to Zhang [58]. Accumulation of proline, soluble sugar, and soluble protein was measured according to Sun [59]. The chlorophyll content was detected according to Jin [60].

### 4.7. Histochemical Detection of Reactive Oxygen Species (ROS)

Nitroblue tetrazolium (NBT) and diaminobenzidine (DAB) staining was measured according to Shi [61]. The standard steps were as follows: chrysanthemum leaves were completely immersed in 10 mm phosphate buffer (pH = 7.8) containing 1 mg/mL NBT or DAB at room temperature. The leaves were not placed in 95% ethanol for decolorization until the spots appeared. After that, the sample was observed, and photos of the sample were taken. Finally, H_2_O_2_ and O_2_^−^ concentration were determined by detection kits (Nanjing Jiancheng Bioengineering Institute, Nanjing, China).

### 4.8. Expression of Salt Stress Response Genes in Dgwrky2 Transgenic Chrysanthemum

To evaluate the expression of abiotic stress-related genes, RNA from the WT and transgenic lines was extracted for reverse transcription. Transgenic chrysanthemum stress-responsive gene expression was detected by qRT-PCR. The abiotic stress-response genes monitored were *Cu/ZnSOD*, *CAT*, *APX*, *P5CS*, *DREB1A*, and *DREB2A*. All relevant primers used in the study are listed in Table 1.

### 4.9. Statistical Analysis

All experiments were performed three times for biological repetition to avoid all types of error. All data were analyzed by SPSS version 24.0 (International Business Machines Corporation, Armonk, NY, USA). A one-way analysis of variance, Tukey’s multiple range test (*p* < 0.05), was employed to identify the treatment means to avoid static errors.

## 5. Conclusions

In summary, this study demonstrated that *DgWRKY2* could positively regulate salt stress tolerance. To alleviate the damage of salt stress to plants, *DgWRKY2* overexpression improved expression of stress-related genes, resulting in relatively enhanced photosynthetic capacity, greatly increased activities of antioxidant enzymes, and high accumulation of proline, soluble sugar, and soluble protein. This indicates that *DgWRKY2* may enhance the sensitivity to salinity by enabling antioxidant and osmotic adjustment capabilities. Overall, this study identified *DgWRKY2* as a potential genetic resource for plant salt tolerance. Not only did *DgWRKY2* play an important role in supplementing and perfecting chrysanthemum-tolerant germplasm resources, but it could also be used as a reserved gene for salt-tolerant plant breeding.

## Figures and Tables

**Table 1:** Primers and their sequences in experiment.

Primer	Sequence (5′-3′)
*DgWRKY2*	F: ATTTGTCAAACTTCTCCTCTCTTCT
	R: GTGGGGGTGGGGGTGGATA
*EF1a*	F: TTTTGGTATCTGGTCCTGGAG
	R: CCATTCAAGCGACAGACTCA
*Cu/Zn SOD*	F: CCATTGTTGACAAGCAGATTCCACTCA
	R: ATCATCAGGATCAGCATGGACGACTAC
*CAT*	F: TACAAGCAACGCCCTTCAA
	R: GACCTCTGTTCCCAACAGTCA
*APX*	F: GTTGGCTGGTGTTGTTGCT
	R: GATGGTCGTTTCCCTTAGTTG
*P5CS*	F: TTGGAGCAGAGGTTGGAAT
	R: GCAGGTCTTTGTGGGTGTAG
*DREB1A*	F: CGGTTTTGGCTATGAGGGGT
	R: TTCTTCTGCCAGCGTCACAT
*DREB2A*	F: GATCGTGGCTGAGAGACTCG
	R: TACCCCACGTTCTTTGCCTC

## References

[1] Jamil A., Riaz S., Ashraf M., Foolad M.R. (2011). Gene Expression Profiling of Plants under Salt Stress. Crit. Rev. Plant Sci..

[2] Munns R. (2002). Comparative physiology of salt and water stress. Plant Cell Environ..

[3] Bing L., Zhao B.C., Shen Y.Z., Huang Z.J., Ge R.C. (2008). Progress of Study on Salt Tolerance and Salt Tolerant Related Genes in Plant. J. Hebei Norm. Univ..

[4] Tuteja N. (2007). Mechanisms of high salinity tolerance in plants. Methods Enzymol..

[5] Eulgem T., Rushton P.J., Robatzek S., Somssich I.E. (2000). The WRKY superfamily of plant transcription factors. Trends Plant Sci..

[6] Zhang Y., Yu H., Yang X., Li Q., Ling J., Wang H., Gu X., Huang S., Jiang W. (2016). CsWRKY46, a WRKY transcription factor from cucumber, confers cold resistance in transgenic-plant by regulating a set of cold-stress responsive genes in an ABA-dependent manner. Plant Physiol. Biochem..

[7] Bakshi M., Oelmüller R. (2014). WRKY transcription factors: Jack of many trades in plants. Plant Signal. Behav..

[8] Tripathi P., Rabara R.C., Rushton P.J. (2014). A systems biology perspective on the role of WRKY transcription factors in drought responses in plants. Planta.

[9] Guo Y., Cai Z., Gan S. (2004). Transcriptome of Arabidopsis leaf senescence. Plant Cell Environ..

[10] Qin Y., Tian Y., Liu X. (2015). A wheat salinity-induced WRKY transcription factor TaWRKY93 confers multiple abiotic stress tolerance in Arabidopsis thaliana. Biochem. Biophys. Res. Commun..

[11] Nam T.N., Le H.T., Mai D.S., Tuan N.V. (2017). Overexpression of NbWRKY79, enhances salt stress tolerance in Nicotiana benthamiana. Acta Physiol. Plant..

[12] Du C., Zhao P., Zhang H., Li N., Zheng L., Wang Y. (2017). The Reaumuria trigyna transcription factor RtWRKY1 confers tolerance to salt stress in transgenic Arabidopsis. J. Plant Physiol..

[13] Zhu D., Hou L., Xiao P., Guo Y., Deyholos M.K., Liu X. (2018). VvWRKY30, a grape WRKY transcription factor, plays a positive regulatory role under salinity stress. Plant Sci..

[14] Powers S.K., Lennon S.L., Quindry J., Mehta J.L. (2002). Exercise and cardioprotection. Curr. Opin. Cardiol..

[15] Jiang M., Zhang J. (2002). Water stress-induced abscisic acid accumulation triggers the increased generation of reactive oxygen species and up-regulates the activities of antioxidant enzymes in maize leaves. J. Exp. Bot..

[16] Meloni D.A., Oliva M.A., Martinez C.A., Cambraia J. (2003). Photosynthesis and activity of superoxide dismutase, peroxidase and glutathione reductase in cotton under salt stress. Environ. Exp. Bot..

[17] Moradi F., Ismail A.M. (2007). Responses of photosynthesis, chlorophyll fluorescence and ROS-scavenging systems to salt stress during seedling and reproductive stages in rice. Ann. Bot..

[18] Negi N.P., Sharma V., Sarin N.B. (2017). Pyramiding of Two Antioxidant Enzymes CuZnSOD and cAPX from Salt Tolerant Cell Lines of Arachis hypogeae Confers Drought Stress Tolerance in Nicotiana tabacum. Indian J. Agric. Biochem..

[19] Hui Y., Qiang L., Park S.C., Wang X., Liu Y.J., Zhang Y.G., Tang W., Kou M., Ma D.F. (2016). Overexpression of CuZnSOD, and APX, enhance salt stress tolerance in sweet potato. Plant Physiol. Biochem..

[20] Yang Z., Zhou Y., Ge L., Li G., Liu Q., Xu Y., Jiang L., Yang Y., School of Agriculture, Jiangxi Agricultural University, School of Sciences, Jiangxi Agricultural University (2018). Expression of Cucumber CsCAT3 Gene under Stress and Its Salt Tolerance in Transgenic Arabidopsis thaliana. Mol. Plant Breed..

[21] Guerzoni J.T.S., Belintani N.G., Moreira R.M.P., Hoshino A.A., Domingues D.S., Filho J.C.B., Vieira L.G.E. (2014). Stress-induced Δ1-pyrroline-5-carboxylate synthetase (P5CS) gene confers tolerance to salt stress in transgenic sugarcane. Acta Physiol. Plant..

[22] Wang W., Vinocur B., Altman A. (2003). Plant responses to drought, salinity and extreme temperatures: Towards genetic engineering for stress tolerance. Planta.

[23] Qin F., Kakimoto M., Sakuma Y., Maruyama K., Osakabe Y., Tran L.S., Shinozaki K., Yamaguchi-Shinozaki K. (2010). Regulation and functional analysis of ZmDREB2A in response to drought and heat stresses in *Zea mays* L.. Plant J..

[24] Zhou M.L., Ma J.T., Zhao Y.M., Wei Y.H., Tang Y.X., Wu Y.M. (2012). Improvement of drought and salt tolerance in Arabidopsis and Lotus corniculatus by overexpression of a novel DREB transcription factor from Populus euphratica. Gene.

[25] Ma J.T., Yin C.C., Guo Q.Q., Zhou M.L., Wang Z.L., Wu Y.M. (2014). A novel DREB transcription factor from Halimodendron halodendron, leads to enhance drought and salt tolerance in Arabidopsis. Biol. Plant..

[26] Akça Y., Samsunlu E. (2012). The effect of salt stress on growth, chlorophyll content, proline and nutrient accumulation, and k/na ratio in walnut. Am. Bank..

[27] Wu Y.H., Wang T., Wang K., Liang Q.Y., Bai Z.Y., Liu Q.L., Jiang B.B., Zhang L. (2016). Comparative Analysis of the Chrysanthemum Leaf Transcript Profiling in Response to Salt Stress. PLoS ONE.

[28] Liu Q.L., Xu K.D., Pan Y.Z., Jiang B.B., Liu G.L., Jia Y., Zhang H.Q. (2014). Functional Analysis of a Novel Chrysanthemum WRKY Transcription Factor Gene Involved in Salt Tolerance. Plant Mol. Biol. Rep..

[29] Liu Q.L., Zhong M., Li S., Pan Y.Z., Jiang B.B., Jia Y., Zhang H.Q. (2013). Overexpression of a chrysanthemum transcription factor gene, DgWRKY3, intobacco enhances tolerance to salt stress. Plant Physiol. Biochem..

[30] Wang K., Wu Y.H., Tian X.Q., Bai Z.Y., Liang Q.Y., Liu Q.L., Pan Y.Z., Zhang L., Jiang B.B. (2017). Overexpression of DgWRKY4 Enhances Salt Tolerance in Chrysanthemum Seedlings. Front. Plant Sci..

[31] Liang Q.Y., Wu Y.H., Wang K., Bai Z.Y., Liu Q.L., Pan Y.Z., Zhang L., Jiang B.B. (2017). Chrysanthemum WRKY gene DgWRKY5 enhances tolerance to salt stress in transgenic chrysanthemum. Sci. Rep..

[32] Diao M., Ma L., Wang J., Cui J., Fu A., Liu H. (2014). Selenium Promotes the Growth and Photosynthesis of Tomato Seedlings Under Salt Stress by Enhancing Chloroplast Antioxidant Defense System. J. Plant Growth Regul..

[33] Ben K.R., Abdelly C., Savouré A. (2012). Proline, a multifunctional amino-acid involved in plant adaptation to environmental constraints. Biol. Aujourdhui.

[34] Chaleff R.S. (1980). Further characterization of picloram tolerant mutance of *Nicotinana tabacum*. Theor. Appl. Genet..

[35] Wang F., Liu P., Zhu J. (2004). Effect of magnesium (Mg) on contents of free proline, soluble sugar and protein in soybean leaves. J. Henan Agric. Sci..

[36] Fu Q.T., Yu D.Q. (2010). Expression profiles of AtWRKY25, AtWRKY26 and AtWRKY33 under abiotic stresses. Hereditas.

[37] Qin Y.X. (2012). Salt-Tolerant Drought-Tolerant Wheat Gene TaWRKY79 and Application Thereof.

[38] Tian Y.C., Qin Y.X. (2013). Wheat Salt-Tolerant and Drought-Resistant Gene TaWRKY80 and Application Thereof.

[39] Wang H., Hao J., Chen X., Hao Z., Wang X., Lou Y., Peng Y., Guo Z. (2007). Overexpression of rice WRKY89 enhances ultraviolet B tolerance and disease resistance in rice plants. Plant Mol. Biol..

[40] Zhou Q.Y., Tian A.G., Zou H.F., Xie Z.M., Lei G., Huang J., Wang C.M., Wang H.W., Zhang J.S., Chen S.Y. (2008). Soybean WRKY-type transcription factor genes, GmWRKY13, GmWRKY21, and GmWRKY54, confer differential tolerance to abiotic stresses in transgenic Arabidopsis plants. Plant Biotechnol. J..

[41] Wang F., Hou X., Tang J., Wang Z., Wang S., Jiang F., Li Y. (2012). A novel cold-inducible gene from Pak-choi (*Brassica campestris*, ssp. *chinensis*), BcWRKY46, enhances the cold, salt and dehydration stress tolerance in transgenic tobacco. Mol. Biol. Rep..

[42] Song Y., Jing S.J., Yu D.Q. (2009). Overexpression of the stress-induced OsWRKY08 improves osmotic stress tolerance in Arabidopsis. Chin. Sci. Bull..

[43] Zhong G.M., Wu L.T., Wang J.M., Yang Y., Li X.F. (2012). Subcellular localization and expression analysis of transcription factor AtWRKY28 under biotic stresses. J. Agric. Sci. Technol..

[44] Zhao Y., Zhou Y., Jiang H., Li X., Gan D., Peng X., Zhu S., Cheng B. (2011). Systematic Analysis of Sequences and Expression Patterns of Drought-Responsive Members of the HD-Zip Gene Family in Maize. PLoS ONE.

[45] Raghavendra A.S., Padmasree K., Saradadevi K. (1994). Interdependence of photosynthesis and respiration in plant cells: Interactions between chloroplasts and mitochondria. Plant Sci..

[46] Li P., Song A., Gao C., Wang L., Wang Y., Sun J., Jiang J., Chen F., Chen S. (2015). Chrysanthemum WRKY gene CmWRKY17, negatively regulates salt stress tolerance in transgenic chrysanthemum and Arabidopsis plants. Plant Cell Rep..

[47] Wang Y., Jiang L., Chen J., Tao L., An Y., Cai H., Guo C. (2018). Overexpression of the alfalfa WRKY11 gene enhances salt tolerance in soybean. PLoS ONE.

[48] Skórzyńskapolit E. (2007). Lipid peroxidation in plant cells, its physiological role and changes under heavy metal stress. Acta Soc. Bot. Pol..

[49] Jain M., Mathur G., Koul S., Sarin N. (2001). Ameliorative effects of proline on salt stress-induced lipid peroxidation in cell lines of groundnut (*Arachis hypogaea* L.). Plant Cell Rep..

[50] Tang M., Liu X., Deng H., Shen S. (2011). Over-expression of JcDREB, a putative AP2/EREBP domain-containing transcription factor gene in woody biodiesel plant Jatropha curcas, enhances salt and freezing tolerance in transgenic Arabidopsis thaliana. Plant Sci. Int. J. Exp. Plant Biol..

[51] Sakuma Y., Maruyama K., Osakabe Y., Qin F., Seki M., Shinozaki K. (2006). Functional analysis of an Arabidopsis transcription factor, DREB2A, involved in drought-responsive gene expression. Plant Cell.

[52] Mallikarjuna G., Mallikarjuna K., Reddy M.K., Kaul T. (2011). Expression of OsDREB2A, transcription factor confers enhanced dehydration and salt stress tolerance in rice (*Oryza sativa*, L.). Biotechnol. Lett..

[53] An G., Watson B.D., Chiang C.C. (1986). Transformation of Tobacco, Tomato, Potato, and Arabidopsis thaliana Using a Binary Ti Vector System. Plant Physiol..

[54] Xue J.P., Yu M., Zhang A.M. (2003). Studies on callus induced from leaves and plantlets regeneration of the traditional Chinese medicine Chrysanthemum morifolium. China J. Chin. Mater. Med..

[55] Schmittgen T.D. (2001). Analysis of relative gene expression data using real-time quantitative PCR and the 2(-Delta Delta C(T)) Method. Methods.

[56] Chen L., Chen Y., Jiang J., Chen S., Chen F., Guan Z., Fang W. (2012). The constitutive expression of Chrysanthemum dichrum ICE1 in Chrysanthemum grandiflorum improves the level of low temperature, salinity and drought tolerance. Plant Cell Rep..

[57] Li H.S. (2015). Principles and Techniques of Plant Physiological and Biochemical Experiment.

[58] Zhang L., Tian L.H., Zhao J.F., Song Y., Zhang C.J., Guo Y. (2009). Identification of an apoplastic protein involved in the initial phase of salt stress response in rice root by two-dimensional electrophoresis. Plant Physiol. Plant Signal. Behav..

[59] Sun H.J., Wang S.F., Chen Y.T. (2009). Effects of salt stress on growth and physiological index of 6 tree species. For. Res..

[60] Jin Y., Donglin L.I., Ding Y., Wang L. (2011). Effects of salt stress on photosynthetic characteristics and chlorophyll content of Sapium sebiferum seedlings. J. Nanjing For. Univ..

[61] Shi J., Fu X.Z., Peng T., Huang X.S., Fan Q.J., Liu J.H. (2010). Spermine pretreatment confers dehydration tolerance of citrus in vitro plants via modulation of antioxidative capacity and stomatal response. Tree Physiol..

